# Strategies to Maintain Skilled Nursing Facility Staffing during COVID: Administrator Perspectives

**DOI:** 10.1093/geroni/igab046.3733

**Published:** 2021-12-17

**Authors:** Amy Meehan, Renee Shield, Caroline Madrigal, Joan Brazier, Emily Gadbois

**Affiliations:** 1 Brown University, Providence, Rhode Island, United States; 2 Providence VA Medical Center, Providence VA Medical Center, Rhode Island, United States

## Abstract

As COVID-19 has resulted in a skilled nursing facility (SNF) staffing crisis, administrators attempt to maintain adequate staffing and stem decreasing patient census levels. We conducted four repeated interviews to date (n=130) at 3-month intervals with administrators from 40 SNFs in eight diverse healthcare markets across the United States. We used thematic analysis to examine their perspectives over time, including the perceived impact on staffing. Results include: 1) the impact of COVID-19 on staffing levels, and 2) strategies used in response to this crisis. Staffing levels have decreased throughout the pandemic, and struggles to maintain adequate staffing levels and patient census numbers have continued as the pipeline of potential new staff constricts. Facilities turned to agencies, many for the first time. Since agencies offer higher salaries, staff are drawn away from employment by SNFs, leading to a cycle of wage wars, and agencies are also challenged to provide staff. SNF administrators describe their responses to this crisis, such as flexible schedules, increased paid time off, sharing of non-direct-patient-care tasks, financial incentives (referral, sign-on, “no-call out”, and other general bonuses); wage analyses, and enhanced employee benefit packages. Some hire recruitment specialists, collaborate with nearby administrators, use creative advertising, or work with local schools. The vaccine mandate worries administrators; as one stated: “I can't afford to lose one person, let alone 20 because of this mandate...”. Given the dwindling pool of potential employees, we present NH administrators’ strategies to attract and retain staff.

